# The Role of Coherent Robot Behavior and Embodiment in Emotion Perception and Recognition During Human-Robot Interaction: Experimental Study

**DOI:** 10.2196/45494

**Published:** 2024-01-26

**Authors:** Laura Fiorini, Grazia D'Onofrio, Alessandra Sorrentino, Federica Gabriella Cornacchia Loizzo, Sergio Russo, Filomena Ciccone, Francesco Giuliani, Daniele Sancarlo, Filippo Cavallo

**Affiliations:** 1 Department of Industrial Engineering University of Florence Firenze Italy; 2 The BioRobotics Institute Scuola Superiore Sant'Anna Pontedera (Pisa) Italy; 3 Clinical Psychology Service, Health Department Fondazione IRCCS Casa Sollievo della Sofferenza San Giovanni Rotondo (Foggia) Italy; 4 Innovation & Research Unit Fondazione IRCCS Casa Sollievo della Sofferenza San Giovanni Rotondo (Foggia) Italy; 5 Complex Unit of Geriatrics Department of Medical Sciences Fondazione IRCCS Casa Sollievo della Sofferenza San Giovanni Rotondo (Foggia) Italy

**Keywords:** social robot, emotion recognition, human emotion perception, human-robot interaction, robot cospeech gestures evaluation

## Abstract

**Background:**

Social robots are becoming increasingly important as companions in our daily lives. Consequently, humans expect to interact with them using the same mental models applied to human-human interactions, including the use of cospeech gestures. Research efforts have been devoted to understanding users’ needs and developing robot’s behavioral models that can perceive the user state and properly plan a reaction. Despite the efforts made, some challenges regarding the effect of robot embodiment and behavior in the perception of emotions remain open.

**Objective:**

The aim of this study is dual. First, it aims to assess the role of the robot’s cospeech gestures and embodiment in the user’s perceived emotions in terms of valence (stimulus pleasantness), arousal (intensity of evoked emotion), and dominance (degree of control exerted by the stimulus). Second, it aims to evaluate the robot’s accuracy in identifying positive, negative, and neutral emotions displayed by interacting humans using 3 supervised machine learning algorithms: support vector machine, random forest, and K-nearest neighbor.

**Methods:**

Pepper robot was used to elicit the 3 emotions in humans using a set of 60 images retrieved from a standardized database. In particular, 2 experimental conditions for emotion elicitation were performed with Pepper robot: with a static behavior or with a robot that expresses coherent (COH) cospeech behavior. Furthermore, to evaluate the role of the robot embodiment, the third elicitation was performed by asking the participant to interact with a PC, where a graphical interface showed the same images. Each participant was requested to undergo only 1 of the 3 experimental conditions.

**Results:**

A total of 60 participants were recruited for this study, 20 for each experimental condition for a total of 3600 interactions. The results showed significant differences (*P*<.05) in valence, arousal, and dominance when stimulated with the Pepper robot behaving COH with respect to the PC condition, thus underlying the importance of the robot’s nonverbal communication and embodiment. A higher valence score was obtained for the elicitation of the robot (COH and robot with static behavior) with respect to the PC. For emotion recognition, the K-nearest neighbor classifiers achieved the best accuracy results. In particular, the COH modality achieved the highest level of accuracy (0.97) when compared with the static behavior and PC elicitations (0.88 and 0.94, respectively).

**Conclusions:**

The results suggest that the use of multimodal communication channels, such as cospeech and visual channels, as in the COH modality, may improve the recognition accuracy of the user’s emotional state and can reinforce the perceived emotion. Future studies should investigate the effect of age, culture, and cognitive profile on the emotion perception and recognition going beyond the limitation of this work.

## Introduction

### Background

During the last decade, there has been increasing interest in research on socially assistive robotics aimed at realizing intelligent robotic solutions for health care and social assistance. We experience an evolution of social robot applications; indeed, they moved from the role of *concierge* and *helper* [1] toward the role of *companion* and *therapist* [2,3]. Social robots have the potential to contribute to the greater good of society; indeed, it has been demonstrated that they can support everyday life as companions and the health care system from *logistics* to *assistance and rehabilitation*. Thinking to include social robots in the care chain, they can be used to reduce stress, anxiety, and pain in children [4]; they can be integrated into conventional behavioral and cognitive therapies for both children and adults who struggle with social anxiety [5]; or they can be used to promote mental health [6]. A review by Hung et al [7] showed evidence that Paro robots can reduce negative emotions in patients, promoting a positive mood and improving social engagement. Rossi et al [8] demonstrated that social robots are effective in decreasing stress in children accessing the emergency room. As the complexity of the robot task increases, social robots are required to perform more complex perceptual, cognitive, and interactive functionalities. This is the case in long-term interactions in which robots and users should establish meaningful communication, emotional awareness, and reliable engagement.

In this context, the human-robot interaction (HRI) field has become crucial, and it is now compelling to better understand how humans perceive, interact with, or accept these machines in social and real contexts. Researchers are also debating on defining the factors that can influence the perceived social capabilities and intelligence of a robot [9,10]. De Graaf et al [11] highlighted the significance of the robot’s social capability, emphasizing the importance of 2-way interaction where a robot is expected to respond to humans in a social manner. In addition, De Graaf et al [11] underlined that a social robot should *also display thoughts and feelings* and should be *socially aware of the environment*, among other issues. When a robot failed to perform this 2-way interaction, people were disappointed and experienced a sense of dissonance. In other words, when interacting with a social robot, especially a humanoid robot, we expect to use the same mental structure and social rules that guide us in human-human communication, expecting empathetic interaction because they are perceived as social actors [12].

From a roboticist or engineering point of view, these concepts are translated into the design and development of behavioral models that can guarantee an efficient and reliable 2-way interaction [13,14]; they should *perceive* and *show* emotions (and social norms) and thus be understood by humans with whom they are interacting. The key challenge in this field is to provide robots with cognitive and affective capabilities, developing architectures that allow them to establish empathetic relationships with users, which can foster long-term and meaningful interactions. From an implementation perspective, the design and the deployment of a socially capable social robot comprises 2 essential parts. The first is devoted to designing and implementing a consistent and congruent emotional behavioral architecture that makes the robot react or act to the environment (ie, *display thoughts and feelings*). The capabilities of a user to understand the emotions displayed by a robot have been explored in different settings [15,16]. Examples of actions can include the expression of congruent cues such as facial expressions [17], changes in the color of the eyes, movement of the upper limbs [16,18], or smart navigation strategies [19]. In contrast, the other part is more focused on the robot’s perception of the user’s emotional response to these behaviors [20], with special attention to contextualizing its action and reaction according to the living contexts and habits or preferences of the person with whom it is interacting (ie*, being socially aware of the environment*) [21].

### Related Work on Emotion and Social Robots

The ability of a robot to perceive the nonverbal cues of the user, which convey user emotion and intent, plays a key role in the development of social robots capable of performing meaningful interactions [22,23]. In this sense, humans’ gaze, body posture, cospeech gestures, and facial expressions play a leading role in defining the context of the interaction, helping the robot to correctly classify the experience, and associating it with informative content [21]. The development of such abilities, for a researcher in the field of robotics, translates into the use of multimodal sensor modality and the implementation of several complex algorithms to endow robots with different cognitive and social capabilities. The visual modality is the most commonly used [24] because it can detect nonverbal behaviors that are representative of the emotional state of users without requiring them to wear any external sensor. Alternatively, wearable sensors [25] can be used, also using a multimodal approach, to overcome the problems related to occlusion and low light. Other algorithms or modules were implemented to perform multiperson tracking [26], speech recognition [27,28], and automatic engagement detection [29]. A recent review paper [24] provides a deep insight into the most used methods and approaches.

For the *show* *emotion* part, robots must exploit several channels (ie, auditory, visual, cospeech, and gestures) and mechanisms (eg, body posture, facial expressions, vocal prosody, touch, and gaze) to communicate their “internal emotional status” and intentions authentically and clearly [30]. Thus, the capabilities of a user to understand the emotions displayed by a robot have been explored in several settings [31]. Over the last few years, several attempts have been made using both video-simulated robots and real robots. Guo et al [20] showed participants 5 different emotions using the humanoid robot called Alpha2, and they were asked to rate the perceived emotion using the Self-Assessment Manikin questionnaire (SAM; only valence and arousal dimensions) [32]. In contrast, Barchard et al [33] conducted a web-based study to evaluate the perception of a robot’s social intelligence by showing videos of robot interactions. However, the embodiment and the appearance of social robots play important roles in the perception of the robot; therefore, video-based elicitation could introduce some bias in the analysis of perceived emotion. This is why other research has relied on investigating the emotion perceived during a real HRI. This is the case of Bagheri et al [34], who asked participants to watch 6 performances of America’s Got Talent Show on Pepper’s tablet that are expected to evoke the 6 basic emotions. Rossi et al [35] and Staffa et al [36] relied on movie trailers to evoke emotions. However, they used nonstandard videos, making it challenging to identify the target emotion in a recognized and standardized manner, as the elicited emotion through the video clips is not known a priori, and consequently, it is difficult to define the role of the robot (and its embodiment) in the elicitation process.

Research groups have recently begun to study the effects of multimodal channels on communication. Studies conducted with embodied conversational agents showed that incongruent emotional stimuli (eg, auditory and visual stimuli) can result in adverse consequences on user rating; conversely, congruent stimuli can facilitate the recognition of emotions [37]. Other researchers have also studied the role of nonverbal behavioral cues while interacting with robots. Movie clips showing coherent and incoherent robot behaviors are often used to elicit emotional responses from users with respect to those induced by movie clips [15,16,18,35]. For instance, Rossi et al [16] investigated how an incoherent nonverbal robot’s behavior with respect to the presented emotion can produce a type of humorous effect. Tsiourti et al [18] investigated how contextual incongruence (ie, a robot’s reaction conflicts with the socioemotional context) can confuse the observers, decreasing the accuracy of the perceived emotion. Nevertheless, such a cospeech robot’s behavior was used in addition to a nonstandard method of emotion elicitation, as previously remarked; thus, it is not easy to understand the role of the robot’s behavior with respect to the emotional context. Therefore, it is important to understand how the robot’s nonverbal behavior might shape the human perception of the showed emotion elicited through standard emotionally labeled visual data sets and, at the same time, observe the robot’s emotion recognition accuracy rate. Although previous studies have shown a correlation between the robot’s nonverbal action and perceived emotion, there is a lack of use of standard elicitation modalities.

**Figure 1:**
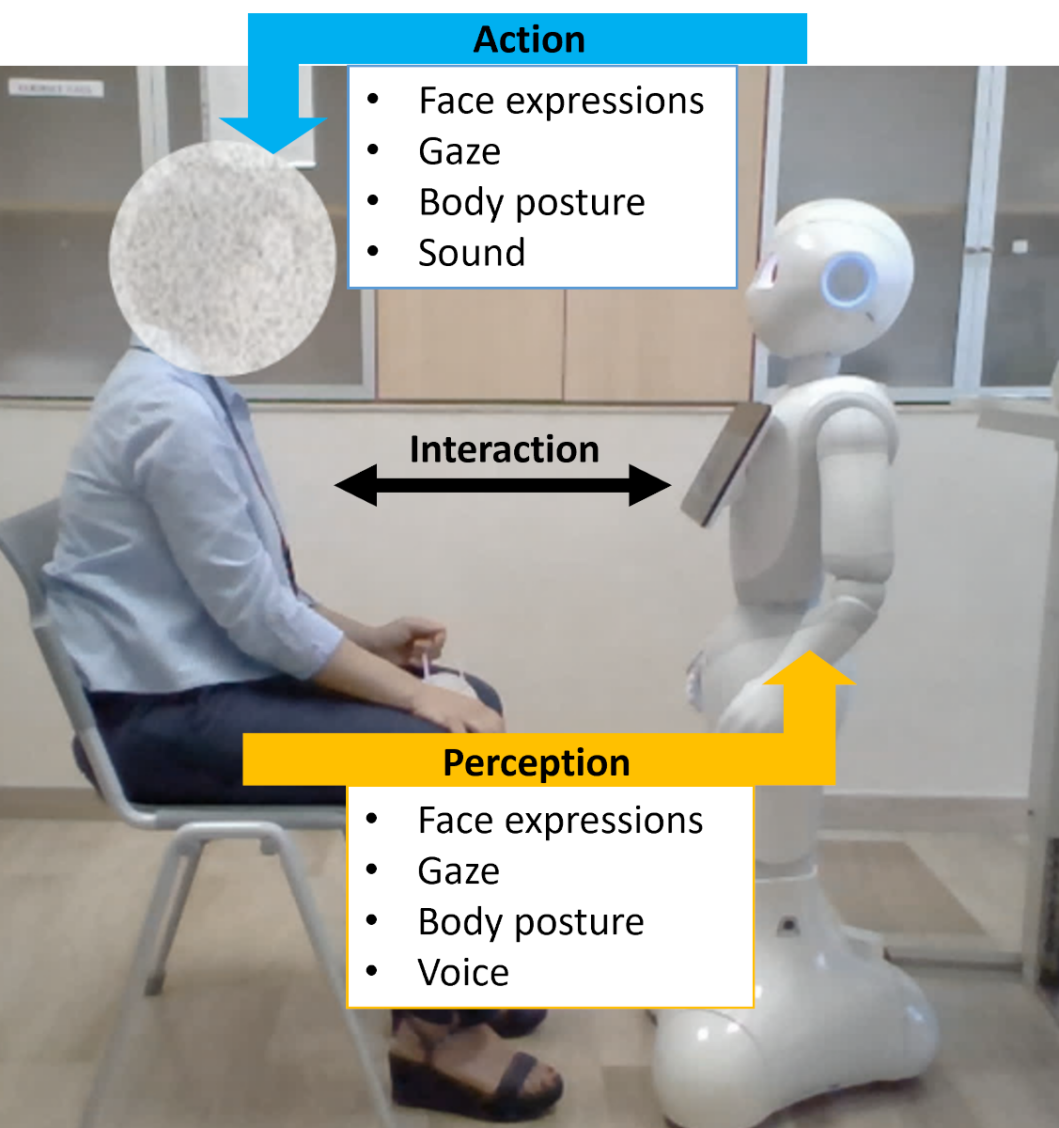
Two-way interaction proposed in this study. To improve the human-robot interaction, the robot should perceive the user’s behavior (yellow arrow) and plan appropriate action (blue arrow).

Therefore, in this work, we present the results of 3 experimental sessions to observe the performance of the robot in recognizing users’ emotions as well as to investigate the difference (if any) in eliciting emotions in humans when using a social robot (with or without coherent behavior) rather than a PC. We plan to use a standard data set of pictures, namely, the International Affective Picture System (IAPS) [38], to elicit emotions in users. Particularly, the robot will use a multimodal behavior (ie, head movements, vocal reinforcement, and body gestures) to interact with the participants while showing the graphical emotions by establishing social binding, whereas the PC will provide emotion elicitation only through a graphical interface. The 2 graphical interfaces have been designed to provide the same information to the user but using different communication channels. In this context, the aim of this work is dual. First, it aims to investigate the increase in the user’s emotional perception during the interaction with a robot with respect to a PC (Figure 1, blue arrow). In particular, this work investigates the role of the robot’s coherent nonverbal behavior in emotion perception by consequently assessing the impact of robot embodiment and, eventually, its coherent behavior. Robot nonverbal cues are manipulated with respect to a mapping between the main associated emotion and cospeech gestures that can be generated on the robot. At the end of each interaction, the participants were asked to self-assess their perceived emotions. In this study, we used the emotion classification proposed by Russel et al [39], which relies on 3 variables, namely, valence, arousal, and dominance. Valence describes the degree to which a stimulus causes a positive or negative emotion, arousal refers to the intensity or level of energy invested in the emotion, and dominance reflects the extent of perceived control over the emotional response when facing the stimulus. The collected answers were analyzed to answer the following research questions (RQs):

1. RQ1: Emotion elicited through a humanoid robot interacting with coherent emotional behavior is rated higher than emotions elicited by a web application in terms of emotional valence, arousal, and dominance.
2. RQ2: There are significant differences in terms of emotional valence, arousal, and dominance between a robot showing coherent behavior rather than a robot that it is not moving at all (static condition).
3. RQ3: The embodiment of the humanoid robot will not affect the emotion perception compared with the web application.

Second, this study aims to assess the accuracy of the robot in recognizing the elicited emotion in the participants (Figure 1, yellow arrow). The ability to infer and interpret emotions plays a key role in establishing intuitive and engaging HRIs. On the one hand, a robot endowed with emotion recognition skills can adapt its behavior based on the detected user emotion [22]. On the other hand, a robot expressing recognizable emotions positively influences the evaluation of its capabilities [40]. In particular, features related to facial expressions were extracted, preprocessed, and analyzed with 3 supervised machine learning techniques to verify the following RQ:

1. RQ4—There is no difference in the robot emotion recognition accuracy despite the elicitation modalities (robot or web application).

In our previous studies [41,42], we evaluated the perceived acceptance and the recognition rate of having a robot that acts coherently and incoherently despite the standard emotion showed with respect to the standard elicitation modality. In contrast, in this study, we focus only on coherent behavior by comparing it with a standard web application that runs on a PC. In addition, instead of focusing on evaluating how the robot’s acceptance is modulated according to the elicitation modality, we focused on the perceived emotion evoked.

## Methods

### Instrumentation

The instrumentation is composed of the following elements: (1) a Pepper robot (Aldebaran, United Robotics Group) or a PC, (2) the RoboMate (Behaviour Labs) interface for cospeech gestures, (3) a custom interface that contains pictures from the IAPS for eliciting emotion, and (4) an external camera placed on Pepper to record the participants’ emotions during the interaction. Pepper is a humanoid robot that is widely used for experimentation in socially assistive robotics. It is 120 cm tall, weighs 28 kg, and has 20 df, including 1 head, 2 arms, and 1 wheeled base. In addition, it has a tablet on the front. Robot coherent behavior was managed through the RoboMate interface [43] to animate Pepper, when necessary, selecting among the behaviors classified as “positive social stimulus” or “negative social stimulus.” The selected stimulus was modeled by a psychologist using 3 modalities: body gestures (upper limb and head), gaze, and sound. IAPS is a database of images devoted to eliciting standardized emotions [44]. It was developed by the Center for Emotion and Attention at the University of Florida. This database is commonly used in psychological studies on emotions and attention. Each image in the data set is labeled with the corresponding emotion, thus enabling researchers to properly select the stimulus. In this study, 60 images were selected from the team of psychologists of the hospital “Casa Sollievo della Sofferenza.” According to the IAPS valence dimension, 21 of the selected images were rated as positive, 19 as negative, and 20 as neutral. A customized web-based interface was developed to standardize the emotional stimulation when using 2 different communication channels (a robot and a PC).

### Experimental Setup

A psychologist welcomed the participant, briefly explaining the experimental setup, including how to use the evaluation tool. It is important to emphasize that the participant was not aware of the real objective of the experimentation, thus avoiding interference with the experience. To properly investigate the RQs, each participant underwent 1 of the following elicitation modalities.

**Figure 2:**
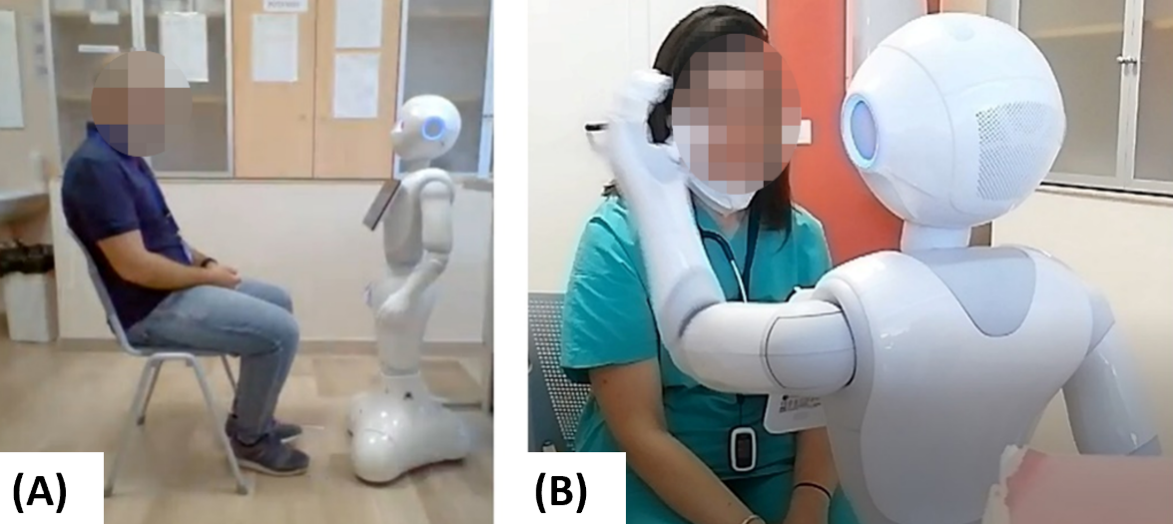
Experimental setup. The participants were interacting with Pepper robot during the experimentation. (A) Participants were asked to sit in front of the robot and watch the images on its tablet. (B) If the participant belonged to the coherent elicitation modality group, the Pepper robot would move its arms, eyes, and head.

1. Static (STA) behavior: Pepper robot has its arms along the body in a neutral position (Figure 2A). Pepper’s face was looking at the participant but without any animacy. Pepper displayed IAPS images on its tablet through the customized web application.
2. Coherent (COH) behavior: Similar to the STA condition, the IAPS images were shown on Pepper’s tablet. Using the RoboMate application, the psychologist assigned a coherent behavior to Pepper with the shown images. In particular, the psychologist can choose and combine 3 modalities for elicited emotions: body gesture (upper limb and head), gaze, and sound, which are available on the RoboMate application (Figure 2B). For example, in the case of positive emotion, Pepper’s gestures were chosen to look friendly; it should look to the user direction, and the voice gave positive reinforcements.
3. PC: For this experimental condition, we used a PC instead of the Pepper robot. Participants were asked to evaluate the images shown on a PC through the customized web application.

The participant was asked to sit in front of the technology (ie, Pepper robot or PC). If the user interacts with Pepper, Pepper is placed 0.5 to 0.6 m far from the user (ie, personal distances [45]); in the case of interaction with the PC, the user is requested to sit and interact with the computer as he or she will commonly do.

Each stimulus was shown for 7 seconds, and at the end, the participant was asked to fill out the SAM [32], as adapted in the study by Gatti et al [46] directly on the robot or on the computer after each picture. SAM is an emotion assessment tool that uses graphic scales, depicting cartoon characters expressing 3 emotional elements (valence, arousal, and dominance). Each participant was asked to rate the domains by selecting an image that corresponded to a score between 1 and 9. A picture of the interface is presented in Multimedia Appendix 1.

At the end of the experimental session, each participant completed 60 SAM questionnaires. The psychologist was present during the test, and she or he was ready to intervene in case of necessity. All the tests were performed at the “Casa Sollievo della Sofferenza” research hospital.

### Ethical Considerations

The approval of the study for experiments using human participants was obtained from the local Ethics Committee on Human Experimentation (register code 3038/01DG). All participants signed an informed consent form before participating in this study, and pictured participants provided written informed consent to allow their image to be published. The data were pseudoanonymized and stored on a GDPR-compliant server.

### Participants

Participants were recruited from July 2020 to February 2021 from employees and staff of the “Casa Sollievo della Sofferenza” research hospital located in Apulia (San Giovanni Rotondo, Foggia) using convenience sampling. Participants were excluded if they had a hearing or visual impairment. Recruited participants were then randomly assigned to undergo 1 of the 3 experimental conditions (ie, STA, COH, and PC). Sociodemographic information (age, education, and sex) was collected to verify the similarities between the groups.

### Data Analysis

#### Overview

Owing to the sample size of each cohort, the nonparametric statistic was used, particularly the Kruskal-Wallis test and chi-square test, to investigate significant differences between participants’ groups in terms of age, sex, and educational level. The significance level was set at *P*=.05. The following paragraphs describe the analysis performed on the SAM questionnaires and the data collected from camera sensors.

#### Emotion Perception Analysis

A total of 60 SAM questionnaires were collected for each participant. The average values of the valence, arousal, and dominance domains were computed for each selected image of each group of elicitation modality (ie, STA, COH, and PC). Differences were analyzed with the Kruskal-Wallis test (*P*<.05) and post hoc evaluated with the Mann-Whitney *U* test (with Bonferroni correction) used to identify between which pair of elicitation modes the difference has occurred.

#### Emotion Recognition Analysis

Data from the camera were processed and examined offline. The recordings were initially analyzed [47] to ensure that only the frames featuring the face of the person performing the test were included in the study. Then the recordings were segmented, providing short videos that corresponded to the user’s reaction to each image proposed, totaling 60 videos per user. The OpenFace toolkit [48] was used to extract 150 features related to gaze and facial expression from each video as well as the quality (ie, confidence) of the extracted features. The data were filtered according to the confidence score (frames with a confidence score <0.90 were discarded). The data were then labeled based on the IAPS-defined emotions (ie, positive, negative, and neutral). Data were normalized and selected. Only features with a correlation coefficient of <0.85 were picked from the initial data set, avoiding those with a high correlation coefficient (which may represent redundant information). The data of the merged data set were then separated into sub–data sets (one for each participant), and emotion classification was performed using the selected features. In this study, we rely on state-of-the-art methods used for emotion recognition [24] to facilitate a comparison with other works. The 3 supervised classifiers used are support vector machine (SVM), random forest (RF), and K-nearest neighbor (KNN). To classify the data by participant, a 10-fold cross-validation procedure was applied, and the outputs were organized in a confusion matrix. The classification performance was assessed in terms of accuracy, precision, recall, and F-measure [49]. The calculations were computed using MATLAB 2020a. More details on emotion recognition analysis are available in Multimedia Appendix 2 [24,47-49].

## Results

### Description of the Participant Cohort

A total of 60 participants were involved in this study, 20 for each modality, resulting in 3600 interactions with technologies. In total, 3 participants were excluded from the analysis of perceived emotion because not all SAM evaluations were correctly saved after each elicitation. In case of missing SAM values, these ratings were removed from the analysis of average values. Finally, 57 participants were included in these subgroups of analyses linked to RQ1, RQ2, and RQ3. Regarding the recognition of emotion using machine learning techniques (linked to RQ4), a total of 53 participants were included in the analysis. A total of 7 participants were excluded because of technical problems related to the quality of the recorded images. The statistical tests did not indicate any difference between the 3 participant cohorts regarding age, sex, and educational level. The participant demographics and educational analyses are reported in Multimedia Appendix 3.

### Participants’ Perceived Emotion Results

**Figure 3:**
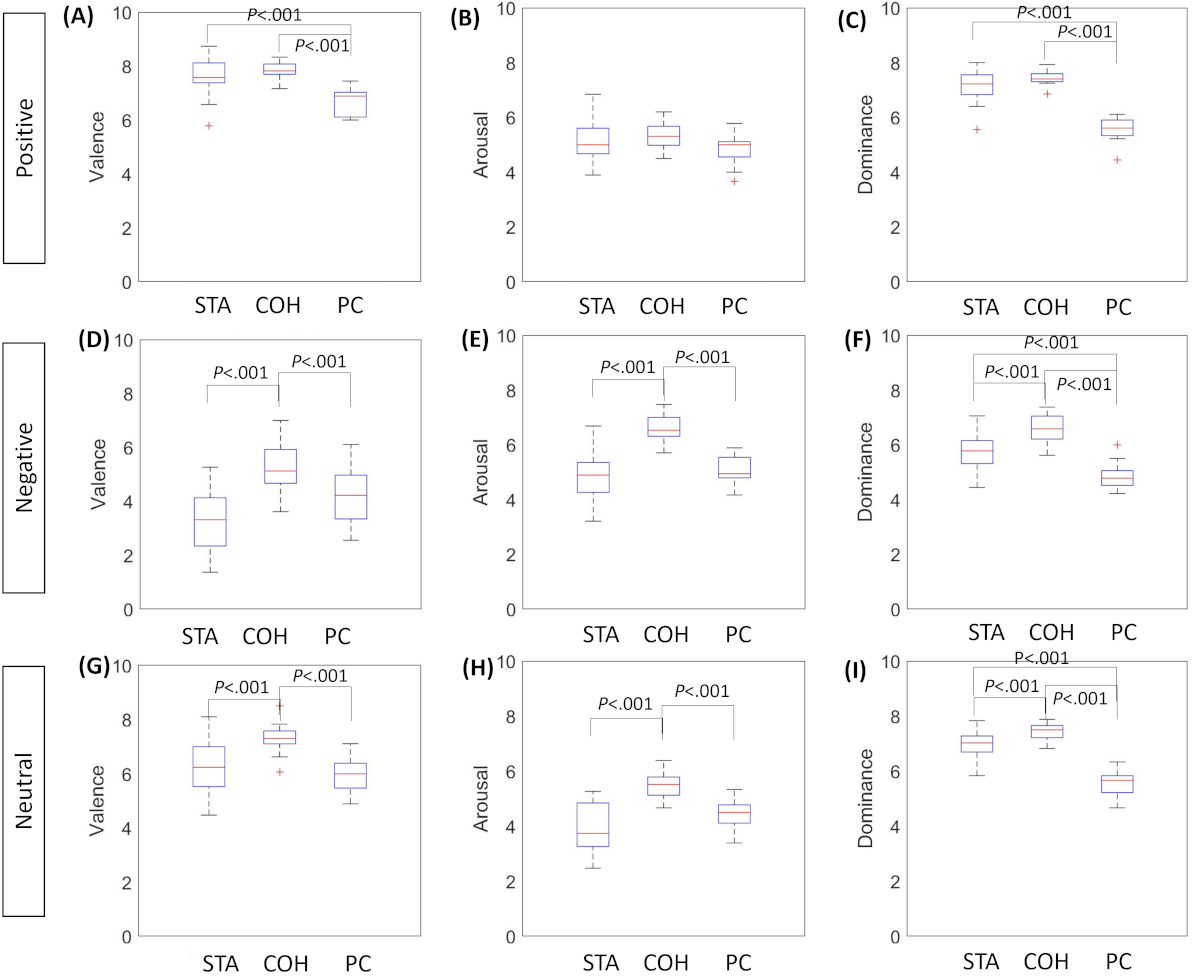
Self-Assessment Manikin Questionnaire results for the 3 elicitation modalities. Boxplot matrix (A), (B), and (C) denote valence, arousal, and dominance for the positive elicitation, respectively; (D), (E), and (F) denote valence, arousal, and dominance for the negative elicitation, respectively; (G), (H), and (I) denote valence, arousal, and dominance for the neutral elicitation, respectively; asterisks on boxplot remark the significant differences evaluated with the Mann-Whitney post hoc test corrected with Bonferroni. COH: coherent; STA: static.

The results underline significant differences (*P*<.001) in the perceived emotions according to the different elicitation modalities, except for the arousal elicited with the positive images (Figure 3). The median and IQR values are fully reported in Multimedia Appendix 4. As for valence, the robot with coherent behavior elicited significant differences (*P*<.001) and higher values in terms of valence, arousal, and dominance domains compared with the other 2 modalities for negative and neutral emotions. In terms of negative valence, the participants perceived fewer negative emotions with the coherent robot than with the other 2 modalities. For positive valence, elicitation with the web application is significantly different from that with the robot (*P*<.001).

Regarding arousal, the coherent robot was rated higher than the other 2 modalities, but there were significant differences (*P*<.001) only for negative and neutral emotions, whereas for positive arousal, the results, depicted in Figure 3, highlight only a trend. All the *P* values are reported in Multimedia Appendix 4.

The participants stimulated using the robot rated significantly higher dominance across all 3 emotions rather than the cohort that used the PC in the test. As for positive elicitation, we found significant differences (*P*<.001) between the cohort stimulated with the PC and those stimulated with the robot (ie, static behavior and coherent behavior). Indeed, the participants rated the emotions (in terms of valence and arousal) elicited by the robot more than the ones elicited using the PC. All *P* values are reported in Multimedia Appendix 4.

### Robot’s Emotion Recognition Results

**Figure 4:**
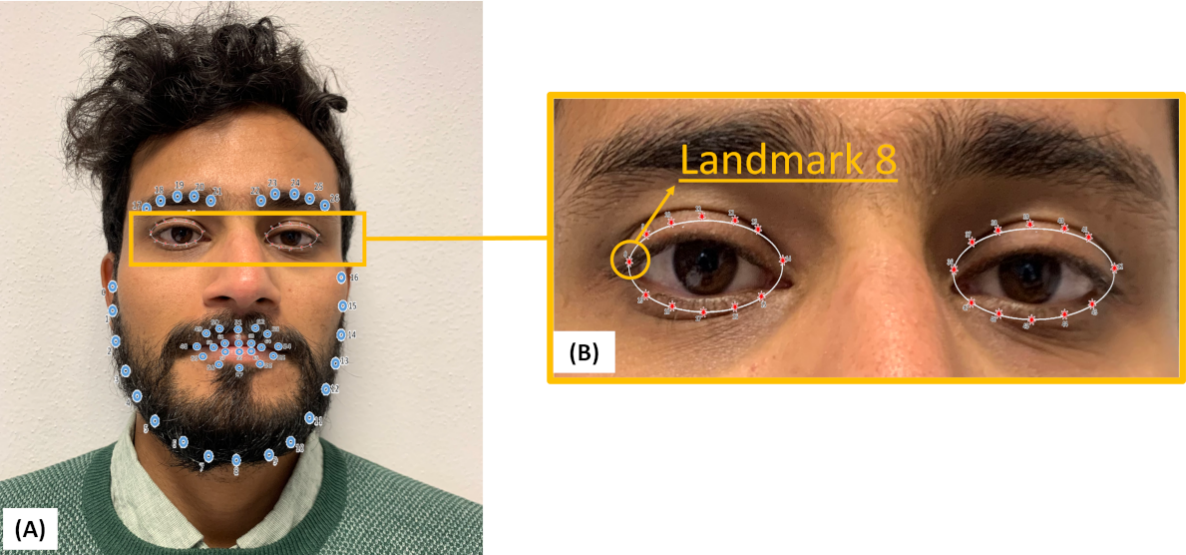
Selected features. (A) Face and (B) eye landmarks extracted with OpenFace software. The landmark 8 in panel B was chosen after the feature selection.

Because of technical issues 1848 frames pertaining to the PC modality were removed from the analysis during the preprocessing. At the end, the total number of samples included in this study was 296,677 for the STA modality, 228,170 for the COH modality, and 103,758 for the PC modality. The number of columns in each data set corresponded to the number of features selected using the correlation analysis method. The following features were selected (Figure 4):

1. The x-, y-, and z-coordinates of the eye gaze direction vector for eye 0 (3 features).
2. The z-coordinate of the eye gaze direction vector for eye 1 (1 feature).
3. The x- and y-coordinates of the location of the landmark 8 (the leftmost in the image) of the eye 0 (2 features).

The 53 data sets were fed into 3 classifiers (SVM, RF, and KNN) [24]. The data sets were uniformly distributed across the 3 groups, as presented in Table 1.

**Table 1:** Distribution of data set instances.

Group	Positive, n (%)	Negative, n (%)	Neutral, n (%)
Static (n=296,677)	103,992 (35.03)	94,257 (31.77)	98,499 (33.2)
Coherent (n=228,170)	70,710 (30.99)	74,383 (32.6)	83,077 (36.41)
PC (n=103,758)	35,195 (33.92)	32,072 (30.91)	36,492 (35.17)

Accuracy, precision, F-measure, and recall were calculated as the mean values from the participants in the same experimental cohort. According to the findings, the KNN classifier offers the best classification results, with an accuracy of up to 0.88 for STA behavior, 0.97 for COH, and 0.94 for PC. The SVM classifiers, in contrast, had the lowest results (accuracy of up to 0.57, 0.67, and 0.68 for STA, COH, and PC, respectively); hence, they were excluded from further research. Compared with the RF classifier, the KNN classifier has the best F-measure (>0.88).

Table 2 presents the complete results for the KNN and RF classifiers, including the accuracy, F-measure, precision, and recall for each group. According to the overall trend, the COH modality achieves a high level of accuracy when compared with the STA and PC elicitations. In terms of the other indicators, the COH was better with the KNN classifier and slightly worse with the RF classifier when it came to elicitation with the PC.

**Table 2:** Performance of K-nearest neighbor (KNN) and random forest (RF) classifiersa.

Group	Accuracy		Precision		F-measure		Recall	
	KNN	RF	KNN	RF	KNN	RF	KNN	RF
Static	0.88	0.65	0.88	0.65	0.88	0.65	0.88	0.65
Coherent	0.97	0.73	0.96	0.72	0.96	0.72	0.96	0.72
PC	0.94	0.74	0.94	0.74	0.94	0.74	0.94	0.74

^a^Mean values are used to calculate the results.

**Figure 5:**
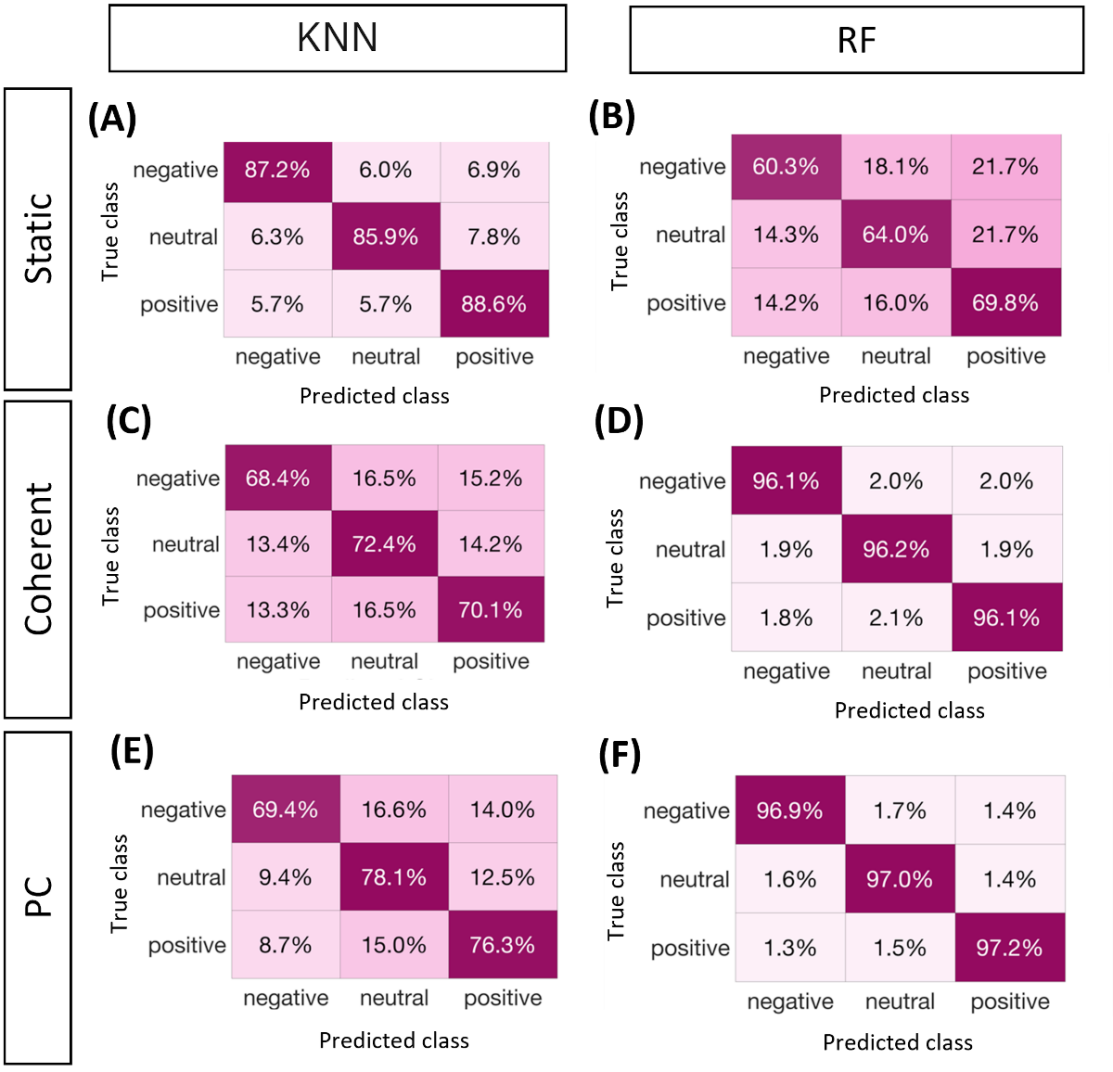
Confusion matrices for K-nearest neighbor (KNN) and random forest (RF) classifiers. The confusion matrices obtained for the 3 elicitation modalities (ie, static, coherent, PC) are reported considering only the KNN and the RF classifiers.

Confusion matrices (Figure 5) for the 3 elicitation modalities were generated to investigate the performance of the classifiers in recognizing the 3 selected emotions. The positive emotion was often better identified, whereas the negative emotion was the least recognized. When the user is stimulated with the robot with coherent modality and the PC, the RF classifier performs better than the KNN classifiers in distinguishing emotions. The KNN classifier appeared to perform better in the static modality than in the other 2.

## Discussion

### Principal Findings

The results confirm RQ1 (“A humanoid robot interacting with coherent emotional behavior is rated higher in terms of emotional valence, arousal, and dominance compared to the web application”) because the COH robot is rated significantly higher for all SAM dimensions (except positive arousal) with respect to the PC condition (Figure 3). However, it is worth noting that when speaking of negative elicitation, receiving a higher rating of valence means that the stimulus with the COH condition was perceived less negatively than the ones elicited with the others. RQ2 (“There are significant differences in terms of emotional valence, arousal, and dominance between the static robot compared to the robot that shows movement”) is confirmed for the 3 dimensions for negative and neutral emotions (Figure 3). It is worth noting that these results confirm that the robot’s movements cause the negative emotion to be perceived as less negative (STA valence median value=3.32; COH valence median value=5.13). As for the positive emotion, there were no significant differences, which could suggest that the robot’s behavior per se did not affect the perception of the positive emotion.

The presented results did not confirm the RQ3 (“The embodiment of humanoid robot will not affect the emotion perception compared to the web application”) for all elicited emotion and SAM constructs. Indeed, there were no significant differences between the STA and the PC elicitation for valence and arousal measured during negative and neutral elicitation (Figure 3). Conversely, COH and STA differed significantly from PC in terms of positive elicitation. These results suggest that robot embodiment per se has a role in the perception of dominance associated with negative and neutral emotions with respect to a standard web interface. On the contrary, as for the positive emotion, embodiment seems to play a key role because both COH and STA elicitations differ from the web application in terms of valence and dominance.

The ability to recognize user emotions is a fundamental step in the development of socially aware robots (RQ4). The emotions were recognized with an average accuracy >0.88 over the 3 elicitation conditions. In addition, the amount of gaze also depends on the interpersonal dynamics between the partner and their personalities and on the intent of using gaze to communicate their internal state. Therefore, it is important to measure it during interactions. As shown in Table 2, the accuracy of COH stimulation was higher than that of the other 2 methods. In addition, the results in the confusion matrices were aligned with the perceived emotion (Figure 5). According to the SAM results, the valence ratings for positive elicitation elicited with PC were significantly different from the other 2 with lower median values. This trend is reflected in the confusion matrices obtained using RF classifiers.

### Comparison With Prior Work

Previous qualitative studies have pointed out how incoherent behavior can generate hilarious reactions in humans [16]. The presented results suggest that we can observe something similar, even if the stimulus is coherent. It appears that the robot’s behavior somewhat distracts from perceiving negative emotions, even if the behavior is aligned with the shown emotion. In addition, as confirmation, positive emotion was perceived significantly more positively than the PC modality, suggesting that robot movements make the robot more positive. Consequently, these results suggest that it is important to tailor the reaction of the robot appropriately to elicit a specific emotion. Indeed, if we need to stimulate—for a certain reason—negatively the users, we need to reduce the robot’s body expression because they can decrease the perception of negative emotions. Alternatively, if we need to provide positive feedback to users, the combined actions of both verbal and nonverbal communication can be used.

A previous study [36] compared robots and web applications that focused on investigating preferences and acceptance, and they did not find any significant deviation in the quantitative results. In contrast, in this study, we focus on human emotion perception, and this perception seems to be influenced or biased by the emotion itself and the robot’s movement. This finding highlights the significance of not just robot embodiment but also its cospeech gestures in designing social agents, particularly when evaluating all dimensions of emotions. Methodologically, the presented findings carry significant implications for the design of experimental protocols. Evaluating HRI cannot rely solely on videos, as they overlook the importance of physical interaction. In the literature, some papers [33] provide a user impression without direct interaction with a robot; the collected results can be biased because the participant missed the contribution in the perception related to embodiment. Take, for instance, the scenario where you are testing a new game application or software on a tablet meant for eventual integration into a robot. Particularly when assessing emotions, it's crucial to approach the generalization of results with caution. In this sense, the result could be altered because the emotions elicited could not be directly applicable when interacting with an embodied agent.

The results obtained for the STA robots with the KNN and RF classifiers were slightly improved with respect to the results obtained in our previous work [42] (average accuracy was equal to 0.85 with KNN and 0.98 with RF), where we used them in combination with encoders. It is also worth noting that after the feature selection process, only the features related to gaze were retained in the analysis. Gaze is extremely important in managing interpersonal interaction and also during human-robot conversation; indeed, it can be correlated with user engagement during conversation or mutual tasks [50,51].

### Limitations of the Study

The limitations of this study were mainly related to the cohort of recruited participants. First, both cognitive and cultural backgrounds are factors that can influence the perception of emotions [52]. Some neurological pathologies (eg, Parkinson disease) can affect facial expressions, whereas others can affect body gestures and language (eg, autism spectrum disorders and apathy); consequently, emotion recognition accuracy in such cases can change. The RQs do not focus on investigating their role in emotion perception; consequently, we recruited cohorts of people comparable for cultural background and cognitive status to limit the impact of these factors. The second limitation of this study refers to how the emotion is evaluated; in this study, we evaluated each SAM dimension separately. The third limitation of this study relies on the supervised machine learning techniques used. In this study, we rely on standard supervised methods because our main RQs are not focused on learning methods; therefore, we apply the most used techniques.

### Future Directions

In this context, by applying the findings and implications of this paper in the health care context, we can conclude that it is important to tailor the reaction of the robot properly; indeed, if we need to stimulate—for a certain clinical reason—the users negatively, we need to reduce the robot’s body expression because they can decrease the perception of negative emotions. Alternatively, if we need to give positive feedback to the users, for instance, during an exercise, we can use the combined action of both verbal and nonverbal communication. To overcome the limitations of this study, future research can be planned to extend the study to include a different group of participants with some cognitive and physical disorders and different cultural backgrounds to evaluate the effect of these factors on emotion perceptions. Future studies should also investigate whether there are differences in combining valence-arousal domains, as proposed in other studies [16,53]. Finally, the data could be analyzed using also deep learning and reinforcement learning techniques.

### Conclusions

This study aimed to investigate the role of robot embodiment and its behavior in emotion perception and recognition using a standard elicitation model. In total, 4 RQs were investigated to understand how the robot’s nonverbal behavior might shape the human perception of the showed emotion elicited through a standard data set and, at the same time, to observe the robot’s emotion recognition accuracy rate. This study presents an experimental setup in which 60 participants were asked to interact with 2 embodied agents (ie, a robot or tablet) that acted as emotion facilitators by showing them 60 standard pictures. The results underline the good recognition accuracy of the perception modules of the robot. Indeed, we can correctly classify the valence of the emotion (ie, positive, neutral, and negative) with an accuracy of up to 0.97 in the best case. According to the results, robot embodiment affects the perception of dominance significantly compared with web applications, which means that participants’ emotions were less controlled when they were interacting with an embodied agent.

## Appendix

Multimedia Appendix 1 Self-Assessment Manikin questionnaire.

Multimedia Appendix 2 Emotion recognition analysis.

Multimedia Appendix 3 Participants’ description.

Multimedia Appendix 4 Median and IQR values computed for each elicited emotion.

## Abbreviations

COH: coherent
HRI: human-robot interaction
IAPS: International Affective Picture System
KNN: K-nearest neighbor
RF: random forest
RQ: research question
SAM: Self-Assessment Manikin questionnaire
STA: static
SVM: support vector machine

## Data Availability

The data sets generated and analyzed during this study are not publicly available because of the scope of the consent signed by the patient participating in the study but are in part (no video recordings) available from the corresponding author on reasonable request.
