# Supplementary material for: The Role of Coherent Robot Behavior and Embodiment in Emotion Perception and Recognition During Human-Robot Interaction: Experimental Study
Source: JMIR Hum Factors. 2024 Jan 26;11:e45494. doi: 10.2196/45494 (PMC10858416; doi:10.2196/45494)

Multimedia Appendix 1 – SAM Questionnaire

SAM Questionnaire proposed by the robot or the web application after showing the picture.

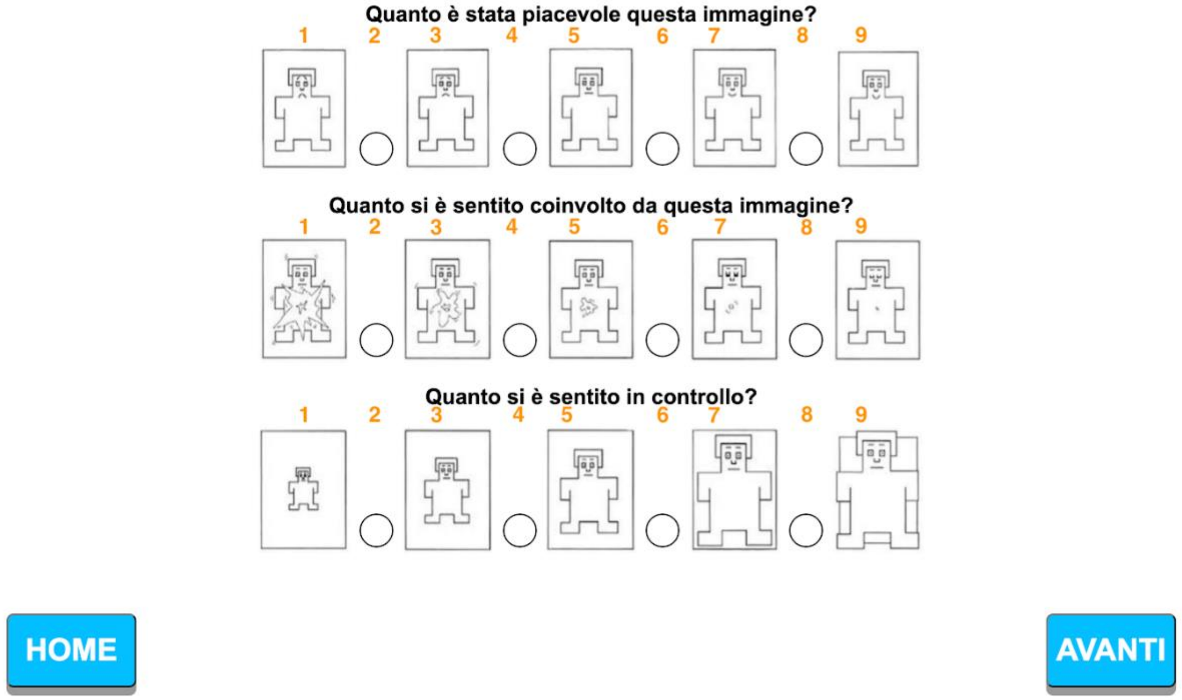

Supplement: Multimedia Appendix 1 [file humanfactors_v11i1e45494_app1.pdf]
