# Supplementary material for: The Role of Coherent Robot Behavior and Embodiment in Emotion Perception and Recognition During Human-Robot Interaction: Experimental Study
Source: JMIR Hum Factors. 2024 Jan 26;11:e45494. doi: 10.2196/45494 (PMC10858416; doi:10.2196/45494)
Supplement: Multimedia Appendix 2 [file humanfactors_v11i1e45494_app2.pdf]

Multimedia Appendix 2 - Emotion Recognition Analysis

The data from the camera were processed and examined offline. The recordings were initially pre-processed with Avidemux software [47] to ensure that only the frames featuring the face of the person performing the test were included in the study. This was done because the camera was occasionally capturing people who assisted the participant but were not taking part in the test.

The pre-processed recordings were then synchronized with the timestamp corresponding to the display of each IAPS image and then segmented, providing short videos that corresponded to the user's reaction to each image proposed, totaling 60 videos per user. The OpenFace toolkit [48] was used to extract 150 features relating to gaze and facial expression from each video, as well as the quality (i.e. confidence) of the extracted features as described in Table 1.

**Feature Number**	**Description**
From feature 1 to feature 3	eye gaze direction vector in world coordinates (x,y,z) for eye 0, the image's leftmost eye
From feature 7 to feature 8	eye gaze direction in radians in world coordinates (x,y) averaged for both eyes
From feature 9 to feature 56	location of 2D eye region landmarks in pixels
From feature 57 to feature 150	location of 2D face region landmark

Table 1 This table describe the features extracted with Open Face.

The data were filtered once these features were extracted. When the face was not well detected from the video, the confidence levels linked with the features obtained by OpenFace software were rather low (e.g., the face was too close to the camera, it was chopped in half, and the individual was wearing spectacles). To ensure the reliability of the derived features, frames with an average confidence of less than 0.90 were eliminated from the study. The data were then labeled based on the IAPS-defined emotions (i.e, positive, negative, neutral).

A normalization step was conducted once the data were labeled (positive, negative, or neutral). In particular, the parameters relating to the positions of the 2D landmarks in pixels were normalized user by user using a Min-Max normalization (Eq. 1).

$$x_{new} = \frac{(x - x_{min})}{(x_{max} - x_{min})}$$

Eq. 1 Min-Max normalization formula.

where x is a set of the current landmark's observed values, and x_{min} and x_{max} are the current landmark's minimum and maximum observed values. The features relevant to the gaze direction had previously been normalized by OpenFace, thus they were left out of the normalization stage.

A feature selection step on the normalized data was used to eliminate redundancy from the detected features. To extract features that are relevant to all the users, we integrated the normalized features of the users into a single dataset. Only features with a correlation

coefficient of less than 0.85 were picked from the initial dataset, avoiding those with a high correlation coefficient (which may represent redundant information). The data of the merged dataset was then separated into sub-datasets (one for each user) and emotion classification was performed using the selected features.

Several supervised classification approaches are used in the literature for emotion recognition, in addition to the innovative enhancements of deep learning and reinforcement learning methodologies. In this work, we rely on state-of-art methods used for emotion recognition [24], indeed three widely used supervised classifiers were used, namely: Support Vector Machine (SVM) with the third-order polynomial kernel, Random Forrest (RF), and K-Nearest Neighbor (KNN). In KNN, the item was simply assigned to the class of that single nearest neighbor, and the distance metric was set to the Euclidean distance. To classify the data by user, a 10-fold cross-validation procedure was applied. Due to the use of well-known benchmark classification techniques in this study, the results are directly comparable to other literary works. The classification performance was assessed in terms of accuracy, precision, recall, and F-measure [49]. The average metrics of each sub-dataset were used to calculate the overall performance for each emotion, and the findings were grouped into confusion matrices to foster the discussion. The calculations were computed in Matlab2020a.
