# Supplementary material for: The Role of Coherent Robot Behavior and Embodiment in Emotion Perception and Recognition During Human-Robot Interaction: Experimental Study
Source: JMIR Hum Factors. 2024 Jan 26;11:e45494. doi: 10.2196/45494 (PMC10858416; doi:10.2196/45494)
Supplement: Multimedia Appendix 3 [file humanfactors_v11i1e45494_app3.pdf]

Multimedia Appendix 3 – Participant Description

Table 1. Participant's characteristics included in the perceived emotion analysis. The p-values were computed with the χ^2 as for the categorical variable of sex and educational level and with the Kruskal-Wallis test for age.

	STA	COH	PC	P
Number of participants (%)	19 (33.3)	19 (33.3)	19 (33.3)	-
Female (%)	13 (68.4)	13 (68.4)	14 (73.8)	.91
Age (IQR)	35 (12.3)	41 (20.7)	45(20.7)	.10
Degree (%)	19 (100)	15 (78.9)	15 (78.9)	.06

Table 2. Participant's characteristics included in the emotion recognition analysis. The P-values were computed with the $\chi 2$ as for the categorical variable of sex and educational level and with the Kruskal-Wallis test for age.

	STA	COH	PC	P
Number of participants (%)	18 (34.0)	19 (35.8)	16 (30.2)	-
Female (%)	11 (61.1)	13 (68.4)	12 (75.0)	.69
Age (IQR)	36.5 (13.0)	41.0 (20.7)	45.0 (17.5)	.18
Degree (%)	17 (94.4)	15 (77.8)	14 (87.5)	.49
