# Supplementary material for: The Role of Coherent Robot Behavior and Embodiment in Emotion Perception and Recognition During Human-Robot Interaction: Experimental Study
Source: JMIR Hum Factors. 2024 Jan 26;11:e45494. doi: 10.2196/45494 (PMC10858416; doi:10.2196/45494)
Supplement: Multimedia Appendix 4 [file humanfactors_v11i1e45494_app4.pdf]

# Multimedia Appendix 4 - Median and IQR values computed for each elicited emotion

**Table 1. Median and IQR values computed for each elicited emotion (i.e. positive, negative and neutral) for each condition (with static robot, with coherent robot and with PC).** The results of the statistical tests were also reported. Codes SC, SP, and CP highlight significant differences accounted by Mann-Whitney post hoc test (with Bonferroni correction) between couples of groups. Namely, SC=difference Static/coherent; SP=difference Static/PC; CP=difference coherent/PC.

| **VALENCE** | | | | | | |
|---|---|---|---|---|---|---|
| **Condition** | **Positive Median (IQR)** | **P-value** | **Negative Median (IQR)** | **P-value** | **Neutral Median (IQR)** | **P-value** |
| **Static robot** | 7.58 | <.001 SP, CP | 3.32 | <.001 SC, CP | 6.24 | <.001 SC, CP |
| **Coherent robot** | 7.82 | | 5.13 | | 7.31 | |
| **PC** | 6.89 | | 4.22 | | 6.00 | |
| **AROUSAL** | | | | | | |
| **Condition** | **Positive Median (IQR)** | **Pvalue** | **Negative Median (IQR)** | **Pvalue** | **Neutral Median (IQR)** | **Pvalue** |
| **Static robot** | 5.00 | - | 4.89 | <0.001 SC, CP | 3.74 | <.001 SC, CP |
| **Coherent robot** | 5.31 | | 6.53 | | 5.51 | |
| **PC** | 5.00 | | 4.94 | | 4.50 | |
| **DOMINANCE** | | | | | | |
| **Condition** | **Positive Median (IQR)** | **Pvalue** | **Negative Median (IQR)** | **Pvalue** | **Neutral Median (IQR)** | **Pvalue** |
| **Static robot** | 7.22 | <.001 SP, CP | 5.78 | <.001 SP, SC, CP | 7.03 | <.001 SP, SC, CP |
| **Coherent robot** | 7.40 | | 6.58 | | 7.50 | |
| **PC** | 5.61 | | 4.78 | | 5.67 | |
